# Noscapine Modulates Neuronal Response to Oxygen-glucose Deprivation/Reperfusion Injury Via Activation of Sigma-1 Receptor in Primary Cortical Cultures

**DOI:** 10.22037/ijpr.2019.112317.13683

**Published:** 2020

**Authors:** Gelareh Vahabzadeh, Nahid Rahbar-Roshandel, Soltan-Ahmad Ebrahimi

**Affiliations:** *Department of Pharmacology, School of Medicine, Iran University of Medical Sciences, Tehran, Iran.*

**Keywords:** Noscapine, NO, Oxygen-glucose deprivation, Cortical culture, Sigma receptor, Intracellular calcium

## Abstract

In the present study, we investigated the effects of noscapine (0.5-2 µM), an alkaloid from the opium poppy (*Papaver somniferum*), on primary murine cortical neurons exposed to 60 min oxygen–glucose deprivation (OGD) in the presence of 5 µM BD-1047, a selective sigma-1 receptor antagonist. The experiments were performed on cortical neurons after 11–16 days of culture. To initiate oxygen–glucose deprivation, the culture medium was transferred to glucose-free DMEM, and placed in a humidified incubation chamber containing a mixture of 95% N_2_ and 5% CO_2_ at 37 °C for 60 min. In order to explore the effect on neurons under oxygen–glucose deprivation in this condition, some cultures were pretreated with noscapine and BD1047 together, 24 h prior to OGD followed by 24 h recovery. Cell viability, nitric oxide (NO) production and intracellular calcium concentration ([Ca^2+^]i) levels were evaluated by MTT assay, the modified Griess method, and Fura-2, respectively. Pretreatment of the cultures with noscapine in the presence of BD1047 significantly increased cell viability and decreased NO generation in a dose-dependent manner compared to BD1047 alone. Pretreatment with 2 μM noscapine and BD-1047 was shown to decrease the rise in [Ca^2+^]i induced by sodium azide (NaN3) and glucose deprivation. We concluded that noscapine in the presence of BD1047 could protect primary cortical neurons after oxygen–glucose deprivation-induced cell injury but this effect was not complete. Our results indicate that neuroprotective effects of noscapine could be mediated partially through activation of sigma-1 receptor and by decreasing NO production and [Ca^2+^]i levels.

## Introduction

Excitotoxicity and excessive intracellular calcium play central roles in causing neurodegenerative disease such as stroke and traumatic brain injury, amyotrophic lateral sclerosis, Alzheimer’s disease, and Parkinson’s disease (1, 2). Although in excitotoxicity process, glutamate has an important role in cerebral injury subsequent to stroke, sigma receptor agonists are also effective in reducing *in-vitro* ischemic process by diminishing the glutamate release and blocking the excessive intracellular calcium (2–4).

Initially it was believed that the sigma receptors were a subtype of opioid receptor; however, further studies have shown that these receptors are different, independent classes of proteins and have a distinct pharmacological profile consisting of two subtypes termed sigma-1 and sigma-2 (5–8). Both subtypes of receptors are widely distributed in different parts of the CNS, *e.g. *prefrontal cortex, hippocampus, striatum, and several other tissues, including the immune and endocrine systems (2, 6 and 9).

 These receptors have many physiological and pathophysiological roles in learning and memory, movement disorders, drug addiction, amnesia, pain, depression, Alzheimer’s disease, stroke, retinal neuroprotection, HIV infection, and cancer. So, they are candidate as a therapeutic target for various diseases (3, 10). In this context, especially sigma-1 receptors have an important role in modul-ating several physiological event such as the regulation of inositol 1,4,5-triphosphate receptors and calcium mobilization from endoplasmic reticulum stores, modulation of calcium entry through ion channels at the plasma membrane including Kv1.4 channels, N**-**methyl-d-aspartate (NMDA) receptors, and voltage-dependent calcium channels. Simi-larly, in *in-vitro* and *in-vivo* studies, sigma-1 receptor activation inhibited ischemic injury by decreasing NO production via suppressing such as decrease glutamate and NMDA-induced nitric oxide synthase (NOS) activation. Therefore, the sigma-1 receptor could be a novel target in treatment of many diseases such as stroke (6, 11–14). For example, it has been shown that activation of sigma-1 receptor agonists has protective effect in focal cerebral ischemia injury by suppression neuronal NO production (11). Also, Katnik *et al*. (3) have shown that regulating intracellular calcium by activation of sigma receptors in cortical neurons exposed to ischemia is one of the mechanisms by which these receptors may possibly exert their neuroprotective properties in this condition. They also demonstrated that the sigma-1 receptor antagonist, BD1047 blocked this neuroprotective effect.

Noscapine is a benzylisoquinoline alkaloid from the opium poppy *Papaver somniferum* and unlike many other alkaloids from the opium latex does not have any notable secondary sedative, hypnotic, analgesic, or euphoric properties (15–17). Ye *et al.* (18) showed that noscapine could inhibit murine lymphoid tumors, probably by suppressing microtubules assembly. Also, this agent could cause cell cycle arrest in several human and murine neoplasms including lymphoma, thymoma, melanoma, and breast cancer and induced apoptosis in both *in-vitro* and *in-vivo* study (15, 19). Kamei (20) showed that pretreatment with rimcazole, a specific antagonist of sigma-receptors decreased antitussive effects of noscapine considerably. Ebrahimi (21) showed that the effect of noscapine on cough suppression was probably via interference with the actions of bradykinin. In addition, Mahmoudian *et al.* (22) demonstrated that noscapine decreased inflammatory damage during ischemia in rat pups model of ischemic brain injury. Khanmoradi *et al.* (23) showed that Noscapine protected renal tissue in Wistar rats against ischemia-reperfusion injury by down regulation of the inflammatory mediators. Another study in our lab demonstrated that noscapine significantly increased neuronal cell viability after oxygen-glucose deprivation for 30, 60, and 240 min in murine primary cortical culture probably by re-establishment of calcium homeostasis following a decrease in NO production (24).

In this work, we aim to determine whether sigma receptors are involved in protective effects of noscapine after oxygen–glucose deprivation/recovery (OGD/R). In addition, we want to show if the protective effects of noscapine are dependent on modulation of intracellular calcium and/or NO production.

## Experimental

*Preparation of primary cell cultures*

Timed pregnant mouse was purchased from Iran University of medical science. Under anaesthesia using chloroform, the pregnant mouse was killed by cervical dislocation and mouse embryos (E_15–18_) were euthanized and decapitated. Fetal cerebral cortex was used for primary mice cortical neuron isolation and cultured as described previously (25, 26).

The resulting homogenate was then centrifuged at 300 ×g for 5 min at 20 °C and were seeded in 12-well plates (at a density of 6 × 10^4^) and glass coverslips pre-treated with poly-D-lysine (10 µg/mL; Sigma–Aldrich) for viability evaluation via OGD and chemical OGD, respectively. The cultures were maintained at 37 °C in 100% humidity and in a 95% air and 5% CO_2_ atmosphere. The plating medium consisted of RPMI-1640 supplemented with 10% FBS, 5% horse serum, and 100 IU/mL penicillin and100 µg/ml streptomycin (Sigma-Aldrich, Saint Louis, MS, USA). 

After 24–48 h, to eliminate the very small number of non-neuronal cells, 10 µM cytosine arabinoside (Sigma, St. Louis, MO, USA) was added to medium culture for 3 days. The experiments were performed after 11-16 days *in-vitro*. All experiments were carried out by the Institute of Animal Care Committee at Iran University of Medical Sciences.

*Oxygen–glucose deprivation and drug exposure*

After 11-16 days in culture, noscapine was exposed to Oxygen–glucose deprivation as described previously (27).

The cells were exposed with 5 µM BD1047, the selective sigma-1 antagonist (Sigma-Aldrich, St. Louis, MO, USA) with or without 0.5-2 µM noscapine (Sigma-Aldrich, St. Louis, MO, USA), 24 h before OGD. The current concentration of noscapine was chosen according to our previous research works.

Briefly, to achieve OGD, the cells were treated to OGD medium containing glucose/glutamine-free DMEM without FBS and transferred in an anaerobic chamber with 95% (v/v) N_2_ and 5% (v/v) CO_2_. The anaerobic chamber was kept sealed at 37 °C. After 60 min in OGD, the chamber was opened and the cultures were removed from the anaerobic chamber and returned to a normoxic environment in RPMI-1640 under the standard cell incubator containing 5% CO2, 95% air balance, and 98% humidity at 37 °C for 24 h. 

*Analysis of neuronal cell viability*

For quantitative measurement of neuronal viability, 3-(4, 5-dimethylthiazol-2-yl)-2, 5-diphenyltetrazolium bromide (MTT) assay was added at a final concentration of 0.5 mg/mL for 4 h as previously described by (28). 

After the cell incubation with MTT, the insoluble formazan crystals in the living cells was dissolved in 200 μL DMSO for 15 min. The optical density (OD) was measured at 570 nm with Dynex MMX microplate reader (Dynex, Richfield, MN, USA). Survival of the control group was defined as 100%. OGD control group: the plates exposed to OGD conditions without addition of any drugs. Control group: the plates not exposed to OGD and without addition any drugs. BD1047 group: the plates exposed to OGD conditions with addition of 5 µM BD1047 (the selective antagonist of sigma-one receptors). BD1047 and noscapine group: the plates exposed to OGD conditions with addition of 5 µM BD1047 and different concentrations of noscapine (0.5-2 µM).

*Nitrite analysis*

Detecting NO_2_^–^ in culture supernatants was assessed by using modified Griess reaction (Sigma Chemical Co. St. Louis, MO, USA). The Griess reagent system is based on the chemical reaction which reacts with sulfanilic acid to produce the diazonium ion. A Nitrite Standard reference curve must be prepared for each assay for accurate quantitation of NO2^–^ levels in experimental samples.

After 24 h OGD, the medium in each well was removed and centrifuged at 10,000 ×g for 10 min at 20 °C. Nitrite was determined by mixing 100 µL supernatant with an equal volume of Griess reagent at room temperature. After 10 min, the absorbance at 540 nm was determined on a microplate reader.

*Measurement of intracellular free calcium*

To describe the intracellular calcium levels during ischemia, 4 mM NaN_3_ (Sigma-Aldrich, Saint Louis, MS, USA), was used for induction of chemical OGD in the presence of noscapine with or without BD 1047 and continued throughout the entire experiment.

After 30 min chemical OGD, cortical cell cultures grown on coverslips were washed three times with salt solution containing (in mM): 140 NaCl, 3 KCl, 2.5 CaCl_2_, 1.2 MgCl2, 7.7 glucose and 10 HEPES, pH 7.4 and incubated with 4 µM fura 2/AM at room temperature for 30 min in a dark incubator.

We used an Olympus IX-71 inverted microscope and a cooled charge-couple device (CCD) camera equipped with an automated fluorescence filter changer for recording and subsequent [Ca2^+^]i calculation. Fluorescence emission images of intracellular Fura-2 at 510 nm and change its peak excitation from 340 nm to 380 nm in response to calcium binding. Ratio metric analyzes were carried out by using ImageJ software.

*Statistical analysis*

All values were expressed as the mean ± standard error mean (SEM). statistical analysis of triplicate measurements represented at least three independent experiments to obtain comparable results for statistical analysis. Significant differences among multiple groups were examined using One-way analysis of variance (ANOVA) followed with Dunnett test. Statistical significance was defined as *P*-values less than 0.05 (*P* < 0.05). the data were analyzed using Graph Pad Prism 6.0 software.

## Results

*Involvement of sigma-1 receptors in pro-tection after OGD/R*

To clarify whether the protective effect of noscapine against 60 min OGD/R-induced cell death was associated with sigma receptors, we examined the effects BD1047, the sigma-1 selective antagonist in the presence or absence of different concentrations of noscapine. Cell survival following OGD-induced cell damage in the presence of BD1047 and different concentrations of noscapine was determined.

**Figure 1:**
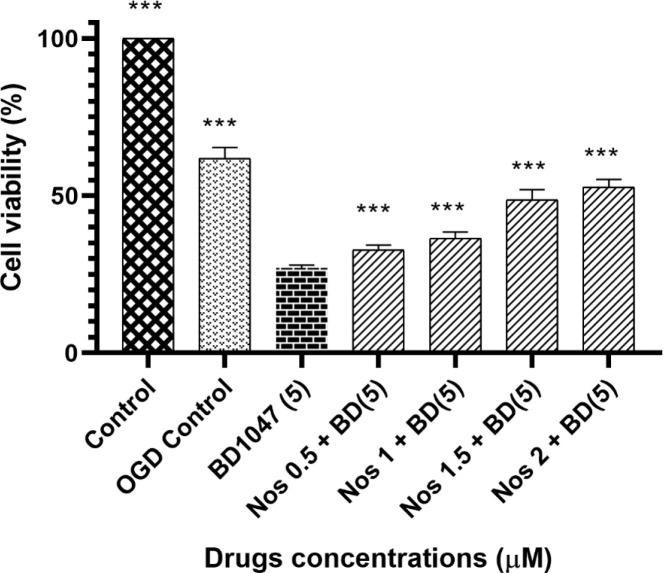
The effect of 5 µM BD1047 in the presence of different concentrations of noscapine on primary cultured murine cortical neurons subjected to 60 min oxygen-glucose deprivation/24 h recovery-induced cell injury. Cell viability was determined using MTT assay. The values are presented as the mean ± SEM. ^***^P < 0.001 *vs.* BD1047 group

As shown in Figure 1 the decrease in cell viability induced by 5 µM BD1047 (27.05% ± 0.32) was statistically significant when compared to the OGD control group (61.92% ± 1.2).

Our results showed that pretreatment of neuronal cells with BD1047 followed by noscapine (0.5-2 µmol) after 60 min oxygen–glucose deprivation/24 h recovery decreased cell viability compared with OGD control group.

However, although noscapine at its highest concentration was unable to exert a complete protective effect (compared to OGD/R control), it did have a concentration dependent protective effect.

*Involvement of sigma-1 receptors in changing NO production after OGD/R*

**Figure 2:**
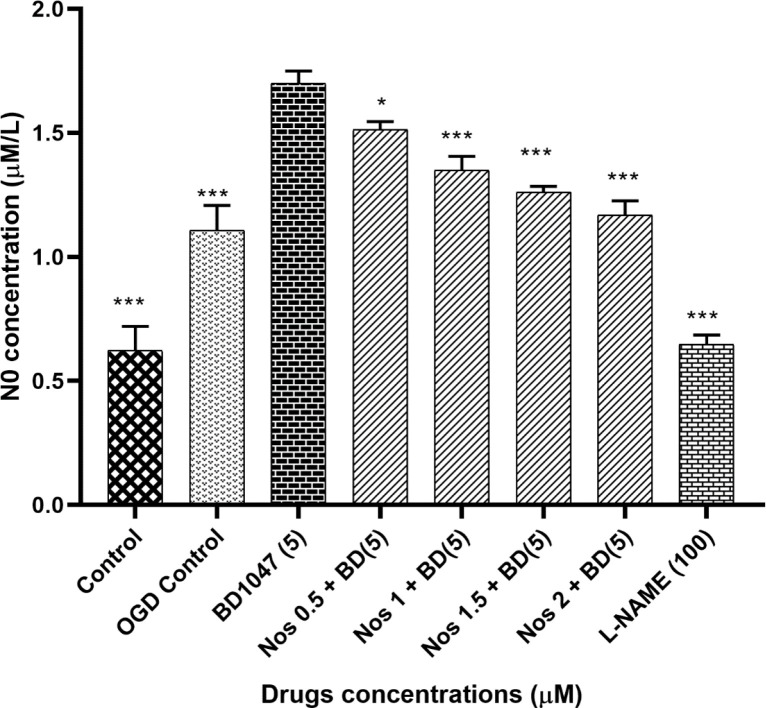
The effect of 5 µM BD1047 on NO production in the presence of different concentrations of noscapine on murine primary cultured cortical neurons during 30 min oxygen-glucose deprivation/24 h recovery-induced cell injury. The values are presented as the mean ± SEM. ^*^P < 0.05, ^***^P < 0.001* vs.* BD1047 group

We examined the effects of 5 µM BD1047 on NO production in primary cortical neurons exposed to 30 min OGD/R in the presence of different concentrations of noscapine. As shown in Figure 2, the pre-treatment of neuronal cells with BD1047 (1.70 ± 0.03) after 30 min OGD/R, produced significantly more nitrite compared to OGD control group (1.11 ± 0.04). Pre-treating neurons with BD1047 and noscapine (0.5-2 µM) together after 30 min OGD/R induced a decrease in NO production as compared with BD1047 alone. The effects of noscapine in the presence of BD1047 on NO production were concentration dependent; however even at the highest noscapine concentration the nitrite levels did not return to OGD/R control group levels.

The effects of L-NAME (100 µM) on NO production were also examined. The results indicated that NO production induced by 30 min of oxygen– glucose deprivation was reduced by L-NAME, a NOS inhibitor (Figure 2).

*Involvement of sigma-1 receptors in modulating intracellular calcium levels after OGD/R*

**Figure 3:**
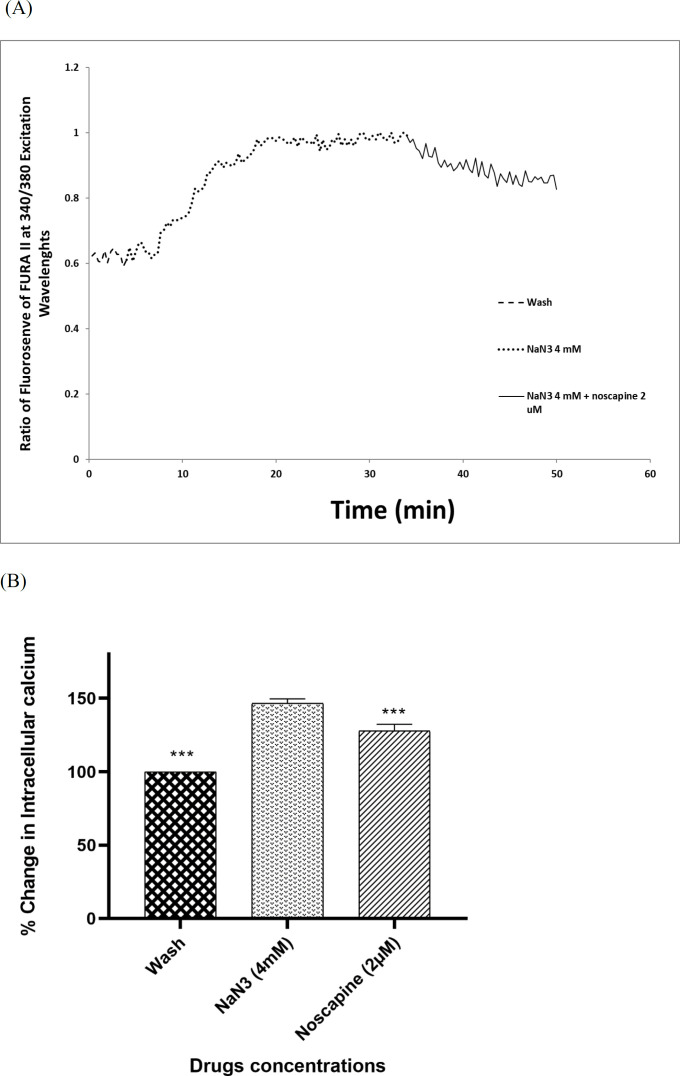
Changes in [Ca^2+^]i levels induced by chemical OGD (4 mM NaN_3_) in the presence of 2 µM noscapine in primary cortical neurons. (A) Time course of NaN_3_-induced [Ca^2+^]i rise in presence of 2 µM noscapine. NaN_3_ (4 mM) stimulated [Ca^2+^]i levels increase. After 30 min ischemic insult, the cells were treated with 2 µM noscapine during chemical ischemia. Two micromolar noscapine could decrease [Ca^2+^]i levels during chemical ischemia. (B) Bar graph of mean change in [Ca^2+^]_i_ levels acquired in response to chemical ischemia in presence of 2 µM noscapine. ^***^P < 0.001* vs.* NaN3 group

**Figure 4:**
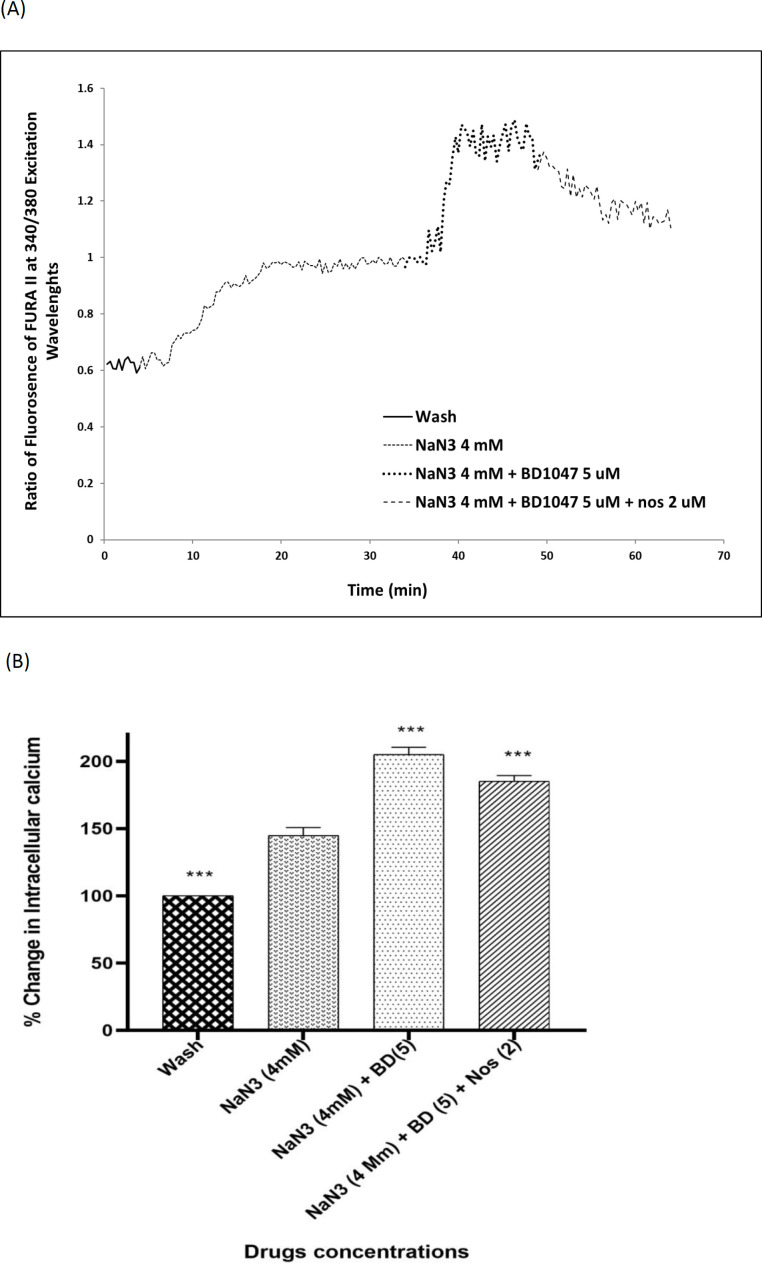
Changes in [Ca^2+^]i levels induced by chemical OGD (4 mM NaN_3_) in the presence of BD1047 with or without 2 µM noscapine in primary cortical neurons. (A) Time course of NaN_3_-induced [Ca^2+^]i rise in presence and absence of 2 µM noscapine and 5 µM BD1047. NaN_3_ (4 mM) stimulated [Ca^2+^]i levels increase. After 30 min ischemic insult, the cells treated with 5 µM BD1047. BD1047 could rise [Ca^2+^]i levels during chemical ischemia. Two micromolar noscapine added to 5 µM BD1047 during ischemia and reduced the [Ca^2+^]i levels compare to BD1047 alone. (B) Bar graph of mean change in [Ca^2+^]_i_ levels acquired in response to chemical ischemia in presence and absence of 2 µM noscapine and 5 µM BD1047. ^***^P < 0.001* vs.* NaN3 group

Four millimolar NaN_3_ was able to induce a significant increase in [Ca^2+^]_i_ levels in cortical neurons bathed in Ca^2+^-containing medium (146.49% ± 1.75) (Figures 3A-3B). Exposure of cortical neurons to NaN_3 _and 2 µM noscapine after 30 min, decreased [Ca^2+^]_i_ levels by 127.73% ± 1.75. However, the chemical OGD-evoked increase in [Ca^2+^]_i_ levels was by 204.98 ± 2.04 in the presence of 5 µM BD1047. Nevertheless, when 2 µM noscapine was used in the presence of 5 µM BD1047 after 30 min, the level of intracellular of calcium declined (185.15% ± 31.48) (Figures 4A-4B).

## Discussion

Research by other workers has demonstrated that glutamate is a key neurotransmitter in the ischemia induced excitotoxicity process. An excess of glutamate is thought to play an important role in the progress of cerebral infarction via NMDA receptor activation that leads to intracellular calcium overload which causes in turn toxic reaction and ultimately leads to cell damage and death (29–31). Although blocking these receptors could help to attenuate neuronal damage, inhibition of other positive effects of NMDA receptors put a limit on usefulness of this treatment. Therefore, other strategies had to be used to treat hypoxic-ischemic brain damage for moderating NMDA receptor function or interaction in the cascade of toxic reactions that led to excitotoxicity. 

According to the previous investigations, sigma receptors could act as putative therapeutic targets for reduction of excitotoxic and neurodegenerative damage (29, 32). Sigma receptors are important because they can greatly affect function of glutamate receptors during excitotoxicity (33). Ligands of both types of sigma receptor subtypes possibly synergistic influenced by several aspects of neurodegeneration and made it possible to modulator effect on neuronal degeneration process far more than traditional drugs (29). More recently a greater role has been attributed to the sigma-1 receptor subtype in some pathological and physiological processes (2). Sigma-1 receptors are located mostly on the endoplasmic reticulum (ER), but when cells are exposed to prolonged stress or their sigma- one agonists, these receptors could translocate to the plasma membrane and modulate different ion channels activities such as voltage-gated and ligand-gated Ca^2+^, K^+^, Na^+ ^and Cl^-^ (34). Therefore, based on these observations it has been suggested that sigma-1 receptor ligands hold promise as protective agents for the treatment of cerebral ischemia (34–36). 

Many studies have shown that sigma receptor ligands have a wide variety of actions including protecting of neuronal cells and treatment of cerebral ischemia in several cells such as retinal ganglion cells, cerebral cortex, primary neuronal cultures, and cells used in ischemic stroke models (29, 33–35 and 37). For example, it has been revealed that the potent sigma-1 receptor ligand 4-phenyl-1-(4-phenylbutyl) piperidine (PPBP) attenuated neuronal injury of primary cortical neuronal cultures when exposed to 120 min OGD or 100 µM glutamate (37). Yang *et al. *(35) have demonstrated that therapeutic use of sigma-1 receptor ligand PPBP protected striatal neurons in newborn piglets model of neonatal global hypoxia-ischemia devoid of showing adverse effects associated with completely blocking NMDA receptors in the developing brain. Similarly, PPBP used in an *in-vitro* model of hypoxia/hypoglycemia in rat primary neuronal cultures and could decrease neurotoxicity caused by cerebral ischemia (32). However, the exact mechanism of this effect is unclear.

In previous studies it has been shown that noscapine could induce the protective effects in variety of ischemic conditions. Khanmoradi *et al.* (23) showed that noscapine exerted renoprotective effects via down regulation of the inflammatory mediators such as tumor necrosis factor-α (TNF-α) and monocyte chemoattractant protein1 (MCP-1) in renal ischemia–recovery injury in rat models. Mahmoudian *et al.* (22) demonstrated that noscapine as a bradykinin antagonist reduced inflammation in a rat model of prenatal hypoxic-ischemic brain edema. Vahabzadeh *et al.* (24) showed that noscapine decreased NO levels and modulation intracellular calcium levels in primary murine fetal cortical. Kamei *et al.* (38) showed that the antitussive effect of noscapine was dose dependently reduced by pre-treatment with rimcazole, a specific antagonist of sigma sites in mice. This is appearing that noscapine has a protective role in ischemic conditions. Therefore, it could be postulated that this protective effect afforded by noscapine were to some extent dependent upon its action on sigma-one receptors. 

As such, the aim of this research is to investigate the effects of noscapine in the presence of 5 µM BD1047, a sigma-1 receptor antagonist in primary cortical culture on 60 min oxygen-glucose deprivation/24 h recovery and show that if different concentrations of noscapine could protect neuronal cells against sigma-1 antagonist in this condition and which mechanisms were involved.

Lack of oxygen and/or glucose is the main reason for ischemic brain injury (39–41). Thus, models have been developed to simulate*, in-vitro* conditions of low or no oxygen/glucose to mimic the pathologic process of ischemic stroke (42,43).

 Goldberg and Choi (44) subjected primary neuronal cultures to no oxygen/ no glucose conditions followed by normal oxygen and glucose exposure. He provided evidence that this model *in-vitro* produced cell damage similar to neurodegeneration that occurs in cerebral ischemic events in man. Therefore, the Goldberg OGD/R model was adopted in the present work.

MTT assay is widely used to measure cell viability and is a good indicator of cellular metabolic activity (45). In this experiment we used MTT assay to measure the number of living cells and were able to show that 5 µM BD1047 did not have neuroprotective effects on primary cortical culture after 60 min OGD, when used alone. However, when cells incubated in 0.5-2 µM noscapine together with 5 µM BD1047 after 60 min OGD/R, the neuroprotective effects of noscapine were increased in a dose-dependent manner compared to BD1047alone but this effect was not complete.

It can be concluded that the neuroprotective effect of noscapine was at least, partially reversed by pretreating cells with BD1047, a selective sigma-1 antagonist. This suggests that activation of sigma-1 receptors is probably involved in the neuroprotective effects of noscapine against OGD-induced cell injury.

During the period of oxygen and/or glucose deprivation, or in the process of nerve damage, amounts of cellular and neuronal energy is diminished; this change results in increase in glutamate and aspartate concentration. Thus, NMDA receptor stimulation, followed by intracellular Ca^2+^ change and Ca^2+^-CaM pathway activation ensue. It is known that excessive calcium in neuron and endothelial cells can activate the Ca^2+-^dependent enzyme cascades such as neuronal and endothelial isoforms of NOS (nNOS and eNOS). Therefore, increased intracellular NO levels after OGD/R, is expected. (30, 46 and 47). In the target cells, NO is converted to the cytotoxic molecule peroxynitrite which causes damage to DNA and therefore leads to cell injury or death (30, 46). In several *in-vitro* and *in-vivo* animal ischemic models, neurotoxicity of NO has been demonstrated. It is also known that under certain pathological conditions, NO production increases *e.g.* during ischemia. This can lead to cell injury and apoptotic cells death by induction of glutamate (11, 35, 46 and 47).

In this work, we were able to show that OGD/R caused an increase in the amount of intracellular NO. Also, our data show that BD1047 enhanced the NO generation due to OGD/R treat. In addition, noscapine was able to partially restore NO levels in the presence of BD1047. 

Many studies have demonstrated that in the presence of ischemia, the generation of NO increases (11, 30, 35, 41 and 48). For example, Goyagi *et al.* (11) showed that PPBP, the potent sigma-1 agonist, had neuroprotective effects and decreased NO production after *in-vivo* striatal tissue damage due to 90 min middle cerebral artery occlusion (MCAO) in Wistar rats. Also, these effects of PPBP disappeared when nNOS was absent or was inhibited. In another research, Yang *et al.* (35) demonstrated that in striatal neurons of newborn piglets exposed to global hypoxia-ischemia, PPBP protected neurons by inhibiting NOS activity that was linked to the decreased coupling of nNOS to postsynaptic density-95 (PSD-95). 

Therefore, the data obtained in this work is consistent with other investigations in so far as it shows the importance of sigma-one receptors in lowering the NO induced by OGD/R treatment. In addition, we were able to show that NO-attenuating effects of noscapine were also dependent, at least partially, on sigma-one receptor function. 

Change in intracellular calcium ion concentration induced by ischemic condition, result to cell death. Increasing intracellular calcium via stimulation of calcium-sensitive ion channels led to disestablishment of plasma membrane function and finally boosted processes such as proteolysis, lipolysis, and the production of reactive oxygen species that caused cell injury and death (3). In this regard, one important mechanism by which sigma-1 receptor ligands protected the neuronal cells in glutamate receptor-mediated excitotoxicity, was modulating intracellular calcium homeostasis and inhibiting ion channel function (2, 3 and 9). For example; it has been shown that in neurocortical culture which was exposed to ischemia, activation of sigma-1 receptors protects the neuronal cells by decreasing in intracellular calcium concentrations (3).

Sodium azide/glucose deprivation model (chemical OGD) has been demonstrated to provoke electrophysiological and neurochemical changes similar to what is observed in the OGD model and could simulate ischemic conditions. This model is preferable to OGD because of producing neurochemical changes more rapidly and reproducibly, therefore making it easier to record the events that are related to changes in [Ca^2+^]_i_ (49). Thus, in this study, sodium azide/glucose deprivation model was used. Our results showed that at the time of chemical OGD, the [Ca^2+^]i was significantly increased compared to the external control condition. This result is consistent with previous reports, which showed that sodium azide could increase neuronal [Ca^2+^]i, to reduced energy production (3, 49). So, we examined the [Ca^2+^]i level in neuronal cells exposed to 2 µM noscapine after 30 min of sodium azide and glucose deprivation treatment by using the Ca+^2^-indicator Fura-2. The results suggested that exposure to 2 µM noscapine was able to reverse the effects of chemical OGD on intracellular Ca^2+^ levels in primary cortical culture. This study also showed that increase in intracellular calcium by 5 µM BD1047, sigma-1 receptor antagonist, was diminished by noscapine, suggesting that noscapine acted as a sigma-1 receptor agonist. Our results support previous observations in other tissues that sigma-1 receptor ligands could modify intracellular calcium ion concentration. Katnik *et al.* (3) demonstrated that 1,3-di-o-tolyl- guanidine (DTG), a sigma receptor agonist, was able to decrease [Ca^2+^]i elevations in cultured cortical neurons from the embryonic rats exposed to chemical OGD. Also when the cells were treated with BD1047, DTG was not able to prevent ischemia induced increases in [Ca^2+^]i. Mueller *et al.* (34) showed that sigma-1 receptors exert neuroprotective effects on retinal ganglionic cells by suppression of calcium signaling through L-type VGCCs. Hayashi* et al.* (50) also showed that in a neuroblastoma-glioma cell line (NG108), sigma-1 receptors modulated calcium signaling. Our data in the line with these works, demonstrate that noscapine has a protective effect on primary cortical culture exposed to sodium azide and glucose deprivation via reduction of intracellular Ca^2+^ levels thorough sigma-1 receptors. Thus, the role of sigma-1 receptor activation by noscapine was confirmed from the antagonism observed by BD1047.

## Conclusion

In conclusion, our data suggests that at least some of the neuroprotective effects of noscapine are due to its agonistic action on the sigma-1 receptor and that neuroprotective effects of noscapine against ischemia involve sigma-1 receptor activations induced suppression of intracellular NO levels and calcium.
